# Assessing the Epigenetic Status of Human Telomeres

**DOI:** 10.3390/cells8091050

**Published:** 2019-09-07

**Authors:** María I. Vaquero-Sedas, Miguel A. Vega-Palas

**Affiliations:** Instituto de Bioquímica Vegetal y Fotosíntesis, CSIC-Universidad de Sevilla, 41092 Seville, Spain

**Keywords:** telomeres, epigenetics, chromatin immunoprecipitation, microscopy, human, cancer

## Abstract

The epigenetic modifications of human telomeres play a relevant role in telomere functions and cell proliferation. Therefore, their study is becoming an issue of major interest. These epigenetic modifications are usually analyzed by microscopy or by chromatin immunoprecipitation (ChIP). However, these analyses could be challenged by subtelomeres and/or interstitial telomeric sequences (ITSs). Whereas telomeres and subtelomeres cannot be differentiated by microscopy techniques, telomeres and ITSs might not be differentiated in ChIP analyses. In addition, ChIP analyses of telomeres should be properly controlled. Hence, studies focusing on the epigenetic features of human telomeres have to be carefully designed and interpreted. Here, we present a comprehensive discussion on how subtelomeres and ITSs might influence studies of human telomere epigenetics. We specially focus on the influence of ITSs and some experimental aspects of the ChIP technique on ChIP analyses. In addition, we propose a specific pipeline to accurately perform these studies. This pipeline is very simple and can be applied to a wide variety of cells, including cancer cells. Since the epigenetic status of telomeres could influence cancer cells proliferation, this pipeline might help design precise epigenetic treatments for specific cancer types.

## 1. The Epigenetic Nature of Human Telomeres Has Remained Controversial

The length of telomeres and the chromatin organization of telomeric regions influence telomeres function [1]. Two main kinds of chromatin organizations are found within the nucleus of eukaryotic cells: euchromatin and heterochromatin. Euchromatin is usually associated with unique sequences that can be transcriptionally active or inactive depending on their epigenetic landscape. Active euchromatin can acquire a close and silenced conformation through the action of the polycomb complexes 1 and 2, which add the repressive H2AK119ub and H3K27me3 epigenetic marks, respectively [2,3]. This polycomb chromatin can be also referred to as facultative heterochromatin. In turn, constitutive heterochromatin, hereinafter referred to as heterochromatin, usually associates with repetitive sequences that are generally silenced [4,5]. Whereas subtelomeric regions in humans are clearly heterochromatic and exhibit enhanced levels of H3K9me3, DNA methylation, and histones hypoacetylation, the epigenetic nature of human telomeres has remained controversial [6,7,8]. This controversy might have been influenced by experimental limitations of the techniques used to study telomere epigenetics. In addition, different human cell lines can have distinct epigenetic marks at telomeres. 

Here, we discuss how subtelomeres and interstitial telomeric sequences (ITSs) might influence studies of human telomere epigenetics. We specially focus on the influence of ITSs and some experimental aspects of the chromatin immunoprecipitation (ChIP) technique on ChIP analyses of telomeres. In addition, we present a specific pipeline to assess the heterochromatic status of human telomeres by ChIP followed by hybridization and sequencing. This pipeline can be applied to the study of multiple epigenetic modifications in a wide variety of human cell types. Although mass spectrometry techniques can be used to analyze telomere epigenetics, to our knowledge, these techniques have not been used to study the epigenetic features of human telomeres, and they are not discussed here [9,10,11].

## 2. Analysis of the Epigenetic Features of Human Telomeres by Microscopy

Since telomeres and subtelomeres cannot be differentiated by conventional microscopy techniques, it is important to consider the influence of subtelomeric regions when the epigenetic features of telomeres are analyzed by microscopy. Co-localization studies have been very useful to determine that shelterin proteins such as TRF1 or TRF2 associate with human telomeres. The combined staining of either of these telomeric proteins by immunofluorescence and of telomeric DNA by fluorescent in situ hybridization (FISH) render high degrees of co-localization [12,13]. In contrast, the combined staining of heterochromatic markers such as H3K9me3 or heterochromatin protein 1 and TRF1 or TRF2 render low co-localization levels [14,15]. Low levels of heterochromatin co-localization with telomeres might reflect transient telomeres heterochromatinization and/or heterochromatinization of only a subset of telomeres. However, low co-localization levels could also be explained by the existence of subtelomeric heterochromatin. In this context, it is worth noting that H3K9me3 mountains that extend 20 to 200 Kbp in length have been described in about one third of the human embryonic stem cell subtelomeres [8]. Thus, one third of the stem cells telomeres should be expected to co-localize with subtelomeric H3K9me3 mountains. In addition, most telomeric regions in humans contain telomere associated sequences (TASs), which are subtelomeric repetitive elements that can extend up to about 200 Kbp from telomeres [16]. If some of these elements were heterochromatic, their corresponding telomeres should be also expected to co-localize with H3K9me3. Even more, since most TASs are longer than telomeres, which extend about 10 Kbp in length, their epigenetic features might be confused with those of telomeres under the microscope [17]. Consequently, microscopy analyses of telomere epigenetics should be carefully interpreted and reinforced with additional studies.

## 3. The Chromatin Immunoprecipitation Technique

The ChIP technique involves the immunoprecipitation of chromatin with specific antibodies and the additional analysis of the immunoprecipitated DNA [18,19,20]. DNA sequences immunoprecipitated with a specific antibody are thought to be associated in vivo with the chromatin feature that it recognizes. These sequences become enriched in the immunoprecipitated DNA samples with regard to the input—not immunoprecipitated DNA samples. Since immunoprecipitation efficiencies, recoveries, and other experimental variables of the ChIP procedure can vary across experiments, it is highly recommended to express enrichment levels as values relative to a specific sequence or to the whole genome if genome-wide ChIP experiments are performed. In both cases, enrichment levels are relative measurements. 

Special care should be taken when repetitive sequences are studied by ChIP because their chromatin features might vary at different genomic loci. ChIP analyses of repetitive sequences are usually performed by hybridization (ChIP-hyb) or sequencing (ChIP-seq). When telomeres are studied, the telomeric ChIP-hyb or ChIP-seq analyses should not be influenced by ITSs. In ChIP-hyb experiments, ITSs might contribute to the signal obtained after hybridization with a telomeric probe. In turn, ITSs might render significant amounts of reads containing telomeric sequences in ChIP-seq experiments. Hence, the influence of ITSs on these analyses should be estimated and, if possible, minimized. Fortunately, the different sequence organizations of telomeres and ITSs can be exploited to minimize ITSs influence [21,22,23,24]. Whereas telomeres are essentially composed of tandem arrays of perfect telomeric repeats, ITSs usually contain short stretches of perfect telomeric repeats interspersed with degenerate repeats. In fact, it is uncommon for ITSs to contain more than four perfect tandem telomeric repeats in organisms such as humans or *Arabidopsis thaliana* [22,24].

In addition to ITSs, the subtelomeric sequences adjacent to telomeres could also influence ChIP-hyb or ChIP-seq analyses of telomeres. In a scenario where telomeres are not heterochromatic but subtelomeres are, the telomere–subtelomere boundaries could be immunoprecipitated by antibodies against heterochromatin. Therefore, telomere analyses could be influenced by this boundary effect. Its magnitude should depend on the lengths of the telomeres and of the immunoprecipitated chromatin fragments. 

## 4. Analysis of the Epigenetic Features of Human Telomeres by ChIP-Hyb

When ChIP-hyb experiments are performed, it is important to know the contribution of ITSs to the hybridization signal obtained. In the case of *Arabidopsis*, this contribution has been estimated in about 70% [25,26]. Hence, in *Arabidopsis*, ChIP-hyb experiments would reveal the mixed epigenetic features of both telomeres and ITSs. This problem was previously addressed by digesting the ChIP samples with Tru9I, a frequently cutting restriction enzyme that recognizes the sequence TTAA. Tru9I readily digests the degenerated repeats of ITSs but not the perfect telomeric repeats of *Arabidopsis* telomeres, which have the sequence TTTAGGG. Thus, after digesting the ChIP samples with Tru9I, telomeres and ITSs can be easily separated by Southern blot. Then, the telomeric signals can be specifically displayed by hybridization with a telomeric probe, as previously reported [21,25].

In humans, the contribution of ITSs to the signal generated after performing ChIP-hyb experiments has not been estimated. However, this contribution should be low for several reasons. First, FISH studies performed with peptide nucleic acid (PNA) telomere probes display the end of human metaphase chromosomes but not internal ITSs [12]. Although these studies support the validity of ChIP-hyb analyses of human telomeres, they should be carefully considered because ChIP-hyb studies are usually performed with DNA probes. Since PNA probes bind more stably to DNA and are more sensitive to mismatches than DNA probes, the telomeres/ITS hybridization ratio displayed by PNA probes is higher than the ratio displayed by DNA probes [27]. Therefore, PNA probes display telomeres more efficiently than ITSs due to the degenerated nature of ITSs. In the case of *Arabidopsis*, ITSs do not to interfere with the analysis of telomere length by FISH in interphase cells when a telomeric PNA probe is used for hybridization [28]. However, *Arabidopsis* ITSs are efficiently detected in FISH and Southern blot experiments using telomeric DNA probes [25,29,30]. Indeed, as mentioned above, *Arabidopsis* ITSs interfere with the detection of telomeres by DNA probes in ChIP-hyb experiments [21,25].

Besides FISH experiments, additional experimental evidence supports that the contribution of ITSs to human ChIP-hyb studies of telomeres should be low. On the one hand, in silico genomic analyses show that ITSs are not very abundant in the human genome, although they can be found in most human subtelomeric regions [16,24,26]. On the other hand, micrococcal nuclease (MNase) digestion experiments reveal that human telomeres have the short nucleosomal spacing characteristic of eukaryotic telomeres [31,32,33]. MNase preferentially cuts the linker internucleosomal DNA. Thus, when bulk chromatin is partially digested with this enzyme and the resulting DNA fragments are resolved on an agarose gel, a nucleosome ladder can be observed after staining with ethidium bromide. This ladder reflects the bulk nucleosomal spacing of the genome. However, if human nucleosome ladders are hybridized with a telomeric DNA probe, the resulting steps of the ladders shorten. Considering that human ITSs are not expected to be associated with short nucleosomes, as previously found in *Arabidopsis* [25], this result supports that ChIP-hyb experiments hybridized with a telomeric DNA probe should mostly reveal the chromatin organization of bona fide human telomeres. Nevertheless, the specific contribution of ITSs could be determined as previously reported [25].

When the epigenetic features of human telomeres are analyzed by ChIP-hyb, the telomeric hybridization signal should be compared with a control signal to estimate relative enrichments values. If the objective is to study whether telomeres are heterochromatic in a specific cell type, repetitive elements such as the α-satellite or the satellites II and III could be hybridized as controls, since these elements have been found to associate with heterochromatic marks [24,34,35,36,37,38]. However, the specific control probes to be used should be carefully selected and validated.

α-satellite monomers have a length of 171 bp and can exhibit a significant degree of sequence divergence. Tandem arrays of these monomers distribute along centromeres and pericentromeric regions, where they have different chromatin organizations. The α-satellite sequences that localize at centromeres fold into a non-heterochromatic chromatin that contains the histone H3 variant CENP-A and drives kinetochores formation. In turn, the pericentromeric α-satellite monomers organize as heterochromatin domains that can influence centromeres function [35,39]. Precise combinations of α-satellite monomers lead to the formation of higher order repeats (HOR) units. These units iterate in tandem hundreds to thousands of times, are characterized by the number of monomers and their order, and localize at specific chromosomes. Considering that the same α-satellite monomers can be found at centromeres and pericentromeric regions, in principle, it is difficult to select the sequences to be used as heterochromatic controls in ChIP-hyb experiments. However, some α-satellite sequences have been found to associate preferentially with pericentromeric heterochromatin. This is the case of the sequences present in the D1Z5 HOR. These sequences hybridize specifically with the pericentromeric region of human chromosome 1 and associate with H3K9me3 in DLD-1 cells [36,40].

Satellites II and III are short-repetitive elements that contain the sequence TCCATT. These elements distribute in tandem along the pericentromeric regions of specific human chromosomes. Therefore, they associate with heterochromatic marks and could be used as heterochromatic controls in ChIP-hyb experiments [24,34,37,38]. In addition, other repetitive elements such as the interspersed transposons could also be used as heterochromatic controls in ChIP-hyb experiments. However, these transposons should be carefully selected because they can lack of specific heterochromatic marks in distinct cell types. Some long terminal repeats (LTR) retrotransposons, such as certain classes of endogenous retroviruses, rely more on H3K9me3 than on DNA methylation for silencing in mouse stem cells [5]. Besides, some non-LTR retrotransposons such as the AluS repeats do not show H3K9me3 enrichment in HL-60 cells [41].

We recommend validating the repetitive elements to be used as heterochromatic controls in human ChIP-hyb studies of telomeres. Their heterochromatic nature could be verified by microscopy, by digestion with methylation-sensitive restriction enzymes, and by ChIP-seq (see below) [24,36,38].

## 5. Analysis of the Epigenetic Features of Human Telomeres by ChIP-Seq

When ChIP-seq experiments are performed to analyze the epigenetic features of telomeres, it is important to minimize the contribution of ITSs to the total number of telomeric reads counted. In this case, the different sequence organizations of telomeres and ITSs can also be exploited. We were previously able to study the epigenetic modifications of human and *Arabidopsis* telomeres independently of ITSs by analyzing multiple genome-wide ChIP-seq experiments. Since ITSs in both organisms usually contain short arrays of perfect telomeric repeats interspersed with degenerated repeats, the reads originated from ITSs do not contain many perfect telomeric repeats organized in tandem. Therefore, we could identify as telomeric reads those that contained four or more perfect tandem telomeric repeats [22,23,24]. When analyzing human telomeres, we considered telomeric reads as those containing five or more tandem repeats [24]. In addition—and for comparison—we determined the number of reads arising from satellites II and III using their consensus sequences [37]. In this way, we were able to analyze multiple ChIP-seq experiments that addressed the genome-wide distribution of 10 epigenetic marks, including H3K9me3, in different cell lines [4].

Our ChIP-seq studies revealed that telomeres are not enriched in the heterochromatic H3K9me3 mark in multiple telomerase-positive human cell lines, including embryonic stem cells. Instead, they exhibit increased levels of euchromatic marks such as H4K20me1 and H3K27ac. In turn, we found that satellites II and III are enriched in H3K9me3 but not in H4K20me1 or H3K27ac. Interestingly, another ChIP-seq analysis using a different set of data also showed that human telomeres are not enriched in H3K9me3 in CD4+ cells [42]. However, the sequence used to identify telomeric reads in this study was not indicated.

Our ChIP-seq analyses also revealed that telomeres in U2OS and LAN6 cancer cells have heterochromatic levels of H3K9me3, similar to those associated with satellites II and III [24,43]. These cells elongate their telomeres by alternative lengthening of telomeres (ALT), which is a recombinational mechanism that involves changes in the DNA sequence and the protein composition of telomeres [44]. Interestingly, the histone methyltransferase SETDB1 was recently found to deposit H3K9me3 in ALT telomeres and to stimulate the ALT phenotype [45]. Therefore, it will be interesting to ascertain whether heterochromatic telomeres are a hallmark of ALT cancer cells.

## 6. Determining the Heterochromatic Status of Telomeres in Specific Human Cell Lines

**Figure 1:**
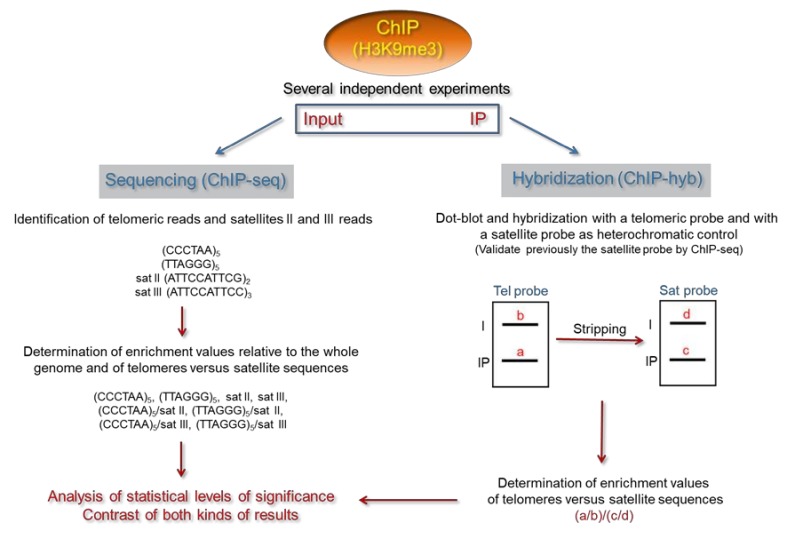
Schematic representation of the experimental pipeline proposed to assess the heterochromatic status of human telomeres by ChIP. ChIP experiments should be performed using antibodies against H3K9me3. At least three independent biological replicates are recommended, thus statistical analyses of enrichments can be performed. Since the consensus sequences of satellites II and III are [(atTCCATTcg)_2_ + (atg)_1–2_]_n_ and [(ATTCC)_7–13_ + (ATTcgggttg)_1_]_n_, respectively, the sequences indicated in the figure could be used to monitor satellites reads in the chromatin immunoprecipitation sequencing (ChIP-seq) experiments [37]. Satellite sequences could also be used as heterochromatic controls in the ChIP-hybridization (ChIP-hyb) studies once they were validated by ChIP-seq. Antibodies against alternative heterochromatic marks such as H4K20me3 or DNA methylation could be used to assess the heterochromatic status of human telomeres. However, our ChIP-seq analyses reveal low (but significant) levels of H4K20me3 enrichment at satellites II and III (our unpublished results), which seems to be in agreement with previously reported analyses of mouse pericentromeric heterochromatin [5,10].

In the following section, we describe a specific pipeline to address the heterochromatic status of human telomeres by ChIP (Figure 1). This pipeline is simple and can be easily applied to a wide variety of human cells. First, ChIP experiments should be performed using antibodies against H3K9me3. Then, these experiments should be analyzed by sequencing and hybridization. ChIP-seq analyses should be performed as previously reported [24]. The frequency of telomeric and satellites II and III reads should be calculated by dividing their counts between the total number of mapped reads, thus making frequency values relative to the whole genome. In addition, the frequency of telomeric reads with regard to satellite reads should be also estimated. Then, enrichment values should be calculated by dividing the immunoprecipitate frequencies between the frequencies of the corresponding input samples. Finally, statistical levels of significance should be determined.

We recommend proceeding with the ChIP-hyb analyses once the heterochromatic nature of satellite sequences has been verified by ChIP-seq (Figure 1). Considering that human ITSs are not expected to influence ChIP-hyb analyses of telomeres, the ChIP-hyb experiments could be achieved by dot-blot followed by hybridization. Dot-blots should be first hybridized with a telomeric probe. Then, they could be stripped and re-hybridized with a satellite probe. For example, an end-labeled satellite III DNA fragment containing the sequence (ATTCCATTCC)_3_ could be used for hybridization. At that point, relative enrichment values of telomeres versus satellite sequences should be calculated by dividing the intensities of the input-normalized telomeric hybridization signals between the intensities of the corresponding satellite signals. These values, which are expected to be similar to those determined by ChIP-seq, should be used to perform statistical analyses.

## 7. Concluding Remarks

The epigenetic modifications of telomeres are usually studied by microscopy or ChIP. However, these studies might be challenged by subtelomeres and/or ITSs. Whereas telomeres and subtelomeres cannot be differentiated by microscopy techniques, telomeres and ITSs might not be differentiated in ChIP analyses. Hence, studies focusing on the epigenetic features of telomeres should be carefully designed and interpreted. It is important to consider the influence of subtelomeric heterochromatin when the heterochromatic status of human telomeres is analyzed under the microscope. In general, these analyses should be reinforced with additional studies. In addition, when the heterochromatic status of human telomeres is analyzed by ChIP followed by hybridization or sequencing, the influence of ITSs should be minimized, and telomeres should be compared with heterochromatic control sequences. Taking into account these considerations, we propose a specific pipeline to assess the heterochromatic status of human telomeres by ChIP (Figure 1). This pipeline is simple and can be easily applied to a wide variety of epigenetic features and human cells.

Current experimental evidence does not support the presence of heterochromatic marks at telomeres in most commonly studied human cell lines (Table 1). However, unlike different types of telomerase-positive cancer cells, certain cancer cells that undergo ALT have enriched levels of telomeric heterochromatin [24,43,45,46]. Even more, telomeric heterochromatin has been reported to stimulate ALT features [45]. Therefore, considering that about 15% of the human cancers undergo ALT, it will be interesting to ascertain whether heterochromatic telomeres are a hallmark of ALT cancer cells, which could be addressed following the pipeline described here. 

In order to sustain continuous proliferation, cancer cells need to maintain their telomeres either by the action of telomerase or by ALT [13,47]. Hence, interfering with any of these two maintenance mechanisms can compromise cancer cells growth and oncogenic processes. It has been proposed that telomeres maintenance mechanisms could be differentially impaired by epigenetic drugs targeting heterochromatin, which are currently being used or tested in a wide variety of cancer treatments [24,43,45,48]. In this context, it is worth mentioning that ALT-positive cancer cells are more sensitive than telomerase-positive cancer cells to trabectedin, an anti-tumor drug that causes R-loop-dependent DNA damage and leads to replication impairment and genome instability [49,50]. R-loops are three-stranded nucleic acid structures in which RNA invades double stranded DNA, generating a DNA:RNA hybrid and the associated single-stranded DNA. These structures can be formed at telomeric regions by telomeric repeats containing RNAs (TERRA), which are transcribed from subtelomeric promoters towards telomeres. Thus, considering that telomeric heterochromatin has been reported to stimulate ALT features including high levels of TERRA, that TERRA contributes to the formation of telomeric heterochromatin, that the levels of TERRA are indeed increased in ALT cells relative to telomerase-positive cancer cells, and that TERRA transcripts can form R-loops in telomeric regions, the aforementioned differential effect of trabectedin could be related to the epigenetic status of telomeres [45,46,51,52,53,54,55,56]. In ALT cancer cells, telomeric heterochromatin could stimulate the formation of R-loops containing TERRA, which in turn would make ALT cells more sensitive to trabectedin than telomerase-positive cancer cells. In addition, since subtelomeric DNA methylation can repress TERRA expression and R-loop formation at telomeric regions, the loss of subtelomeric DNA methylation previously reported in ALT cells could also stimulate trabectedin sensitivity [6,43,51]. Therefore, the heterochromatic status of both telomeres and subtelomeres might influence the output of trabectedin on cancer cells proliferation. In this scenario, the experimental pipeline described here could help design precise epigenetic treatments for specific cancer types.

## Figures and Tables

**Table 1:** Experimental evidence supporting that telomeres are not heterochromatic in most commonly studied [non-alternative lengthening of telomeres (ALT)] human cell lines.

	Evidence	Ref.
1	Immunofluorescence microscopy studies reveal low co-localization levels of the H3K9me3 or the HP1 heterochromatic marks with telomeres, which could be explained by the presence of H3K9me3 and HP1 in subtelomeric heterochromatin.	[8,14,15,16]
2	ChIP-hyb experiments reveal low levels of H3K9me3 at telomeric repeats as compared with subtelomeric sequences.	[57]
3	ChIP-seq experiments show that telomeres are not enriched in H3K9me3 but have enhanced levels of the H3K27ac and H4K20me1 euchromatic marks. In contrast, satellites II and III are enriched in H3K9me3 but not in H3K27ac or H4K20me1.	[24,42]
4	Human telomeres should not be methylated because the human telomeric sequence is TTAGGG, and DNA methylation in mammals mainly circumscribe to the CG context. In agreement with this assumption, DNA methylation is completely absent from *Arabidopsis* telomeres. However, the *Arabidopsis* telomeric sequence, which is TTTAGGG, is methylated by the CHH methylation machinery (where H is A, C, or T) when it is present in interstitial telomeric sequences (ITSs).	[58,59]
5	Super-resolution microscopy shows that the disruption of shelterin proteins can lead to an increase in telomeres volume that has been related to telomeres decompaction and to a 53BP1-dependent telomere clustering. However, telomeres volume is not affected in human cells treated with inhibitors of heterochromatin formation. Therefore, heterochromatin does not seem to affect the protective structure of telomeres as shelterins do.	[60,61,62]
6	Telomeric nucleosomes are shorter and more sensitive to overall micrococcal nuclease digestion than the heterochromatic nucleosomes associated with chromocenters.	[31,63,64,65]
